# Supplementary material for: Low-Temperature Crystal Structures of the Hard Core Square Shoulder Model
Source: Materials (Basel). 2017 Nov 7;10(11):1280. doi: 10.3390/ma10111280 (PMC5706227; doi:10.3390/ma10111280)
Supplement: Supplementary file 1 [file materials-10-01280-s001.pdf]

Low-temperature crystal structures of the hard core square shoulder model: Supplementary Information

Alexander Gabriëlse, Hartmut Löwen, and Frank Smallenburg
Institut für Theoretische Physik II: Weiche Materie,
Heinrich-Heine-Universität Düsseldorf, Universitätsstr. 1, 40225 Düsseldorf, Germany

CRYSTAL PREDICTION WITH SHIFTED BOUNDARY CONDITIONS

Here we introduce a variation on the "floppy box" Monte Carlo (FBMC) crystal prediction approach proposed in Ref. [1, 2]. In the FBMC approach, candidate crystal structures are predicted by performing a Monte Carlo simulation for a small number of particles (typically $N \lesssim 20$). The particles are contained in a periodic simulation box spanned by three vectors that can fluctuate in both length and direction. Due to the small number of particles, the system can sample a large number of crystal phases with differently shaped unit cells in a short time, allowing it to rapidly find entropically and energetically favorable structures. These structures can then be tested for stability using simulations on a larger scale or theoretical approximations.

The efficiency of simulations of small boxes with a variable shape is highly dependent on the degree of distortion of the simulation box. In particular, if the box is flattened in any direction, particles can interact with periodic images of themselves and other particles that are several boxes away. To keep the box distortion low, the floppy box approach typically makes use of a lattice reduction technique [3], which redescribes a given unit cell in terms of a linear combination of its box vectors.

In order to simplify this extra step in the simulation, we propose a variation on the FBMC approach which makes use of shifted boundary conditions. In this shifted-boundary Monte Carlo (SBMC) approach the unit cell is defined by three perpendicular box vectors

$$\begin{aligned} \mathbf{v}_x &= \{L_x, 0, 0\} \\ \mathbf{v}_y &= \{0, L_y, 0\} \\ \mathbf{v}_z &= \{0, 0, L_z\}, \end{aligned} \tag{1}$$

where $L_{x,y,z}$ are the box lengths along the x, y, and z-axis. Periodic boundary conditions are then shifted such that the origin of the next periodic image of the box in the three directions are given by:

$$\begin{aligned} \mathbf{p}_x &= \{L_x, 0, 0\} \\ \mathbf{p}_y &= \{\delta_{xy} L_x, L_y, 0\} \\ \mathbf{p}_z &= \{\delta_{xz} L_x, \delta_{yz} L_y, L_z\}, \end{aligned} \tag{2}$$

where δ_{ij} is the relative shift along the i-axis associated with moving one periodic image in the j-direction. Note that the vectors $\mathbf{p}_{x,y,z}$ span any unit cell shaped like a parallelepiped. In our simulations, we now simply vary the values of $L_{x,y,z}$ and δ_{ij} independently in our Monte Carlo simulation, sampling different crystal structures in a way that is equivalent to the FBMC method. Effectively, the only change we have made thusfar is the requirement that one of the vectors spanning the periodic unit cell lies along the x-axis, and one lies in the xy-plane, eliminating the (irrelevant) rotational freedom of the full unit cell.

However, in this representation, lattice reduction can be done by a simple redefinition of the shifting parameters δ_{ij} such that they each lie between -0.5 and 0.5. In particular, starting from set of δ_{ij}, the reduced shifting parameters are given by

$$\begin{aligned} \delta_{xy}^{\text{red}} &= m(\delta_{xy}) \\ \delta_{xz}^{\text{red}} &= m(\delta_{xz} - [\delta_{yz}]\delta_{xy}) \\ \delta_{yz}^{\text{red}} &= m(\delta_{yz}), \end{aligned} \tag{3}$$

where $[\cdot]$ denotes rounding to the nearest integer, and $m(x) = x - [x]$ is a modulo function which always returns a number between -0.5 and 0.5.

It is important to note that the reduction of the shifting parameters here does not completely prevent the formation of flat boxes, analogous to the lattice reduction in the FBMC approach. For example, particles can still line up into a widely spaced column in which the particles interact with periodic images several boxes away. Following Ref. [1], we prevent this by implementing a minimum axis length for all three directions, requiring that $L_{x,y,z} > L_{\min} = 0.75\sigma$, with σ the size of the hard core of our particles. No angular restrictions are required.

We store our particle coordinates as scaled coordinates $\mathbf{s}$, such that they can be converted to real-space coordinates via

$$\mathbf{r} = s_1 \mathbf{v}_x + s_2 \mathbf{v}_y + s_3 \mathbf{v}_z. \tag{4}$$

As a result, when performing a Monte Carlo move which changes one of the parameters $L_{x,y,z}$, particle positions scale along with the volume change, and we apply the standard Monte Carlo acceptance rule for volume rescaling (see e.g. Ref. [2]). When performing a move which changes one of the shifting parameters δ_{ij}, the volume remains constant, and acceptance is purely based on the change in energy of the system.

To determine which periodic image particles we should take into account when calculating interactions, we make

use of our minimum axis length $L_{\min}$. Under the condition that $-0.5 \leq \delta_{ij} \leq 0.5$, the origin of an image of the simulation box that is n boxes away along the x, y, or z direction is at least a distance $nL_{\min}$ away from the origin of the simulation box. As a result, any particle in this image box is at least a distance $(n-1)L_{\min}$. Hence, for particles with a maximum interaction range $\sigma + \delta$, we at most have to consider image particles up to $\lceil(\sigma+\delta)/L_{\min}\rceil + 1$ boxes away in each direction. We loop over all of these boxes, and determine the actual minimum distance between a point in the image box and a point in the central box. We then take into account image particles in each image box where this distance is less than the interaction range.

We have confirmed that the FBMC and SBMC approaches largely predict the same structures for our square-shoulder systems with interaction ranges $\delta/\sigma = 0.15$ and 0.20. Although the results are not expected to be identical, both methods find the same set of stable zero-temperature structures, and show strong similarity in the frequencies at which different structures are observed.

It is important to note that the modification to the FBMC approach proposed here is neither significantly faster or more effective for the purpose of predicting candidate crystal structures. However, implementation of the SBMC approach avoids the lattice reduction technique and simplifying the constraints for avoiding extreme distortion of the simulation box. As a result, the SBMC approach is considerably easier to implement.

CELL THEORY USING VORONOI CELLS FOR INTERACTING PARTICLES

Cell theory [4, 5] provides a simple route for calculating a mean-field approximation for the free energy of a crystalline solid. In cell theory, the partition function of a single particle in the crystal is approximated by assuming that all other particles are located at their lattice site. The partition function of the particle under consideration is then written as:

$$Q_1 = \frac{V_0}{\Lambda^3}\left\langle \exp\left(-\beta\left[u(\mathbf{r}) - \frac{1}{2}u(\mathbf{r}_0)\right]\right)\right\rangle_{V_0}, \quad (5)$$

where $\beta = 1/k_BT$ with k_B Boltzmann's constant and T the temperature, and $u(\mathbf{r})$ is the energy of the particle at position $\mathbf{r}$. The thermal wavelength Λ has no effect on the phase behavior, and can therefore freely be chosen to equal the particle diameter σ. In the case of hard spherical particles, this partition function reduces to the free volume available to the central particle (divided by Λ^3). This is commonly approximated by assuming that this volume is a polyhedron, formed by taking the Wigner-Seitz cell of the particle, and moving each of its faces inward to a distance $r_i - \sigma$ from the lattice position of the central particle, where r_i is the distance to neighbor i in the perfect lattice [6]. Although this slightly underestimates the free volume available to the central particle, the effects of this approximation are on the same order as the approximations made in cell theory, and the volume of the resulting polyhedron is significantly easier to calculate than the "true" free volume.

Here, we extend this approach to particles interacting with square shoulder repulsions, with hard core diameter σ, shoulder width δ, and shoulder height ϵ. In this case, we consider for each of the N neighbors of the central particle two possible planes delimiting the free volume of the central particle: one at distance σ from the neighbor position (representing its hard-core repulsion), and one at distance $\sigma + \delta$ from the neighbor position (representing its shoulder), as illustrated in Fig. 1a. Clearly, all of the outer planes again form a polyhedron which approximates the free volume of the central particles if the square-shoulder repulsion was absent (blue in Fig. 1b). Similarly, the innermost polyhedron represents the volume $V_\emptyset$ available to the particle where it interacts with no other particles, i.e. its energy $u = 0$. In total, we can construct 2^N polyhedra, each associated with a different combination of particle interactions. For example, the red polyhedron drawn in Fig. 1c shows the volume $V_{\{5\}}$ available to the particle if it *at most* interacts with neighbor 5. We can calculate the subvolume associated with the case where the particle interacts *only* with neighbor 5 (shaded blue in Fig. 1c) by calculating $\delta V_{\{5\}} = V_{\{5\}} - V_\emptyset$. Using the same approach, we can calculate e.g. the subvolume $V_{\{5,6\}}$ (Fig. 1d) where the particle interacts with both particle 5 and 6 via

$$\delta V_{\{5,6\}} = V_{\{5,6\}} - \delta V_{\{5\}} - \delta V_{\{6\}} - V_\emptyset. \quad (6)$$

This approach can be straightforwardly repeated until the subvolume for each set of interactions is known. The total single-particle partition function can then be written as:

$$Q = \sum_{i=1}^{2^N} \delta V_{S_i} \exp(-\beta(u_{S_i} - u_{\text{ref}}/2)), \quad (7)$$

where δV_{S_i} denotes the subvolume for interacting with particles in the set S_i of neighbors, and u_{S_i} is the energy of that state, given by ϵ times the length of S_i. Additionally, u_{ref} is the energy of the particle in the central position.

To calculate these volumes, we construct 2^N polyhedra and calculate their volume V_i using the Voro++ library [7]. Any cell that does not contain the central lattice position is assumed to have volume $V_i = 0$. This only occurs if the ground state energy of the crystal cell under consideration has an energy $u_{\text{ref}} > 0$. Effectively, this means that we ignore the energy change associated with moving away from a neighbor the particle interacts

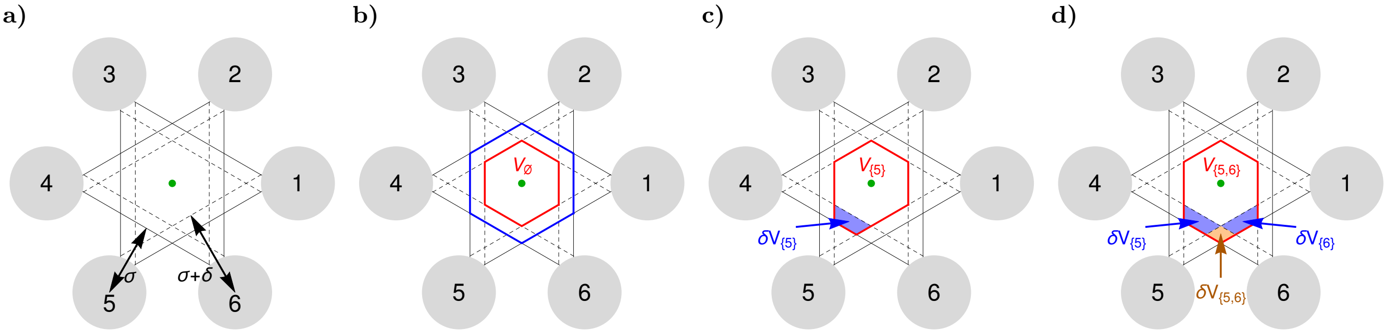

FIG. 1: Two-dimensional illustration of the calculation of the approximate volume of different regions contributing to the cell theory free energy. **a)** Construction of the planes delimiting the different regions. For each neighbor (labeled 1 through 6), we draw two planes: one at distance σ from the neighbor (solid lines), and one at distance $\sigma + \delta$ (dashed lines). **b)** Total volume available in the cell (blue), and the volume $V_\emptyset$ available with no interactions (red). **c)** Determination of the subvolume $\delta V_{\{5\}}$ From $V_\emptyset$ and $V_{\{5\}}$. **d)** Determination of the subvolume $\delta V_{\{5,6\}}$.

with at its ideal lattice site. This additional approximation is justified by the observation that if these positions contribute meaningfully to the free energy, the cell contains local energy minima which do not correspond to the central lattice position, implying that the particle has multiple favored positions within its cell. Since this conflicts with the mean-field assumption that all particles are (on average) at their lattice site, cell theory is already likely to break down in these cases. Moreover, a crystal structure where particles can lower their energy by moving away from their lattice site is not likely to be stable. After calculating all volumes, we calculate the associated subvolumes by subtracting from each volume V_i all subvolumes that correspond to neighbor sets that are strict subsets of neighbor set i. If we set $\delta V_0 = V_0$, we can write this as:

$$\delta V_{S_i} = V_{S_i} - \sum_{j, S_j \subsetneq S_i} \delta V_{S_j}. \tag{8}$$

To test the effect of the additional approximations made in this method, we compared the results of this approach to cell theory free energies calculated using Monte Carlo integration of the single-particle partition function in Eq. 5, performed by repeated insertion of the central particle into a small insertion volume around its central position. The results are in good agreement within the deviations normally expected from cell theory. As an example, we plot in Fig. 2 a comparison of the free energy of a body-centered cubic crystal for interaction range $\delta/\sigma = 0.15$ as a function of density at fixed temperature, calculated using both random insertion and the Voronoi cell approach.

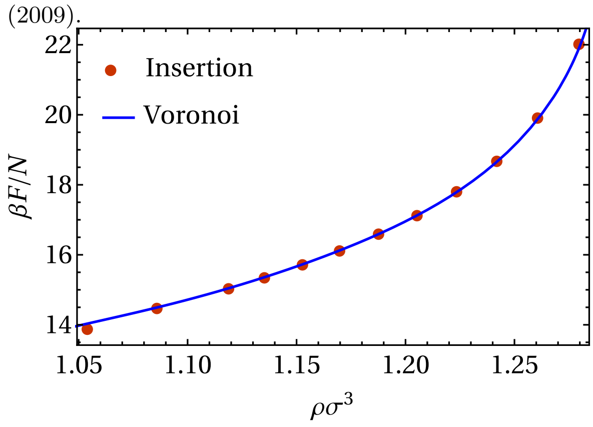

FIG. 2: Comparison between cell theory calculation via direct insertion (points) and via the Voronoi cell approximation (line), for a body-centered cubic crystal at temperature $k_B T/\epsilon = 0.5$ and interaction range $\delta/\sigma = 0.15$.

[1] L. Filion, M. Marechal, B. van Oorschot, D. Pelt, F. Smallenburg, and M. Dijkstra, Phys. Rev. Lett. **103**, 188302 (2009).

[2] J. de Graaf, L. Filion, M. Marechal, R. van Roij, and M. Dijkstra, J. Chem. Phys. **137**, 214101 (2012).

[3] D. Gottwald, G. Kahl, and C. N. Likos, J. Chem. Phys. **122**, 204503 (2005).

[4] S. Prestipino, F. Saija, and G. Malescio, Soft Matter **5**, 2795 (2009).

[5] J. Lennard-Jones and A. Devonshire, Proc. Royal Soc. A 53 (1937).

[6] P. Ziherl and R. D. Kamien, J. Phys. Chem. B **105**, 10147 (2001).

[7] C. H. Rycroft, Chaos **19**, 041111 (2009).
